# Dietary Supplements in Chemotherapy-Induced Peripheral Neuropathy: A New Hope?

**DOI:** 10.3390/nu14030625

**Published:** 2022-01-31

**Authors:** Katarzyna Szklener, Sebastian Szklener, Adam Michalski, Klaudia Żak, Weronika Kuryło, Konrad Rejdak, Sławomir Mańdziuk

**Affiliations:** 1 Department of Clinical Oncology and Chemotherapy, Medical University of Lublin, 8 Jaczewski Street, 20-090 Lublin, Poland; slawomir.mandziuk@umlub.pl; 2 Department of Neurology, Medical University of Lublin, 8 Jaczewski Street, 20-090 Lublin, Poland; sebastianszklener@gmail.com (S.S.); konrad.rejdak@umlub.pl (K.R.); 3 Student Scientific Association, Department of Clinical Oncology and Chemotherapy, Medical University of Lublin, 8 Jaczewski Street, 20-090 Lublin, Poland; michalski.g.adam@gmail.com (A.M.); zakklaudia3@gmail.com (K.Ż.); weronika.kurylo@vp.pl (W.K.)

**Keywords:** neurotoxicity, peripheral neuropathy, chemotherapy, cancer therapy, dietary supplements

## Abstract

Chemotherapy-induced peripheral neuropathy (CIPN) is one of the main and most prevalent side effects of chemotherapy, significantly affecting the quality of life of patients and the course of chemotherapeutic treatment. Nevertheless, despite its prevalence, the management of the CIPN is considered particularly challenging, with this condition often being perceived as very difficult or even impossible to prevent with currently available agents. Therefore, it is imperative to find better options for patients diagnosed with this condition. While the search for the new agents must continue, another opportunity should be taken into consideration—repurposing of the already known medications. As proposed, acetyl-L-carnitine, vitamins (group B and E), extracts of medical plants, including goshajinkigan, curcumin and others, unsaturated fatty acids, as well as the diet composed of so-called “sirtuin-activating foods”, could change the typical way of treatment of CIPN, improve the quality of life of patients and maintain the continuity of chemotherapy. This review summarizes currently available data regarding mentioned above agents and evaluates the rationale behind future research focused on their efficacy in CIPN.

## 1. Introduction

Recent years have proven cancer to be one of the major causes of death worldwide [1]. Due to the advances in medicine, and new diagnostic tools, cancer can be detected sooner and faster and therefore, the introduction of a proper treatment at an earlier stage is possible. Nevertheless, cancer survivors report experiencing a remarkably worse quality of life (QoL), mostly due to the vast array of possible side effects of current anticancer treatments, including pain and symptoms caused by toxicity to the nervous system [2,3]. One of such neurotoxic symptoms of anticancer treatment is chemotherapy-induced peripheral neuropathy (CIPN), a common side effect of chemotherapy, defined as symptoms or signs related to damaged peripheral nerves, either autonomic or somatic [4].

CIPN usually presents itself as a “glove and stocking” neuropathy, although in severe cases, due to the weakness of the long nerves, it may advance proximally thus affecting bigger areas of the limbs [5,6]. It typically presents itself symmetrically, with sensory symptoms ranging from a mild tingling sensation through numbness, to a spontaneous feeling of burning or hypersensitivity, with the most common motor dysfunctions including muscle weakness and impaired balance [7,8,9]. The exact frequency of CIPN is agent dependent, but in the meta-analysis of over 4000 patients, the median prevalence was assessed to be 68.1% after one month after the end of chemotherapy, 60.0% after 3 months and 30.0% after 6 months or more [10]. The medications most frequently associated with the development of CIPN are platinum-based drugs, taxanes, thalidomide and its analogues, vinca alkaloids, bortezomib and ixabepilone [3,11,12]. In most cases, peripheral neuropathy caused by those drugs is late, with its severity being proportional to the cumulative dose of the administered drug; contrary to that, peripheral neuropathy caused by paclitaxel and oxaliplatin is usually immediate, beginning right after or even during the infusion [11]. Furthermore, several risk factors predisposing to the CIPN have been identified, such as obesity, older age, past history of neuropathies, genetic polymorphisms, more advanced stage of cancer, perineural invasion of cancer, as well as alcohol intake, depression, anxiety, stress, and sleep disturbances [13,14,15,16].

Mechanisms by which chemotherapy-induced neurotoxicity translate to the CIPN are complicated and not fully understood. However, axonal degeneration has been found to be a major hallmark of CIPN [17]. Among the CIPN-causing mechanisms we can find transporter-mediated uptake of chemotherapeutics, oxidative stress resulting from the mitochondrial damage, microtubule disruption causing improper axonal transport, damage to the dorsal root ganglia sensory neurons, abnormal activity of Aδ and C nerve fibres, upregulation of proinflammatory cytokines, changes to ion conductance, and inhibition of growth factors [17,18,19]. Furthermore, altered expression of the voltage-gated ion (Na_v_^+^, K_v_^+^ and Ca_v_^2+^) channels and transient receptor potential (TRP) channels caused by chemotherapy results in the hyperexcitability of the neurons, thus making it a potential cause of the CIPN [19,20]. Moreover, recent reports suggest that inflammation and neuronal physiological functioning disorder caused by the chemotherapy-induced IGF-1 downregulation and the abnormal activation of astrocytes are among the potential mechanisms involved with the development of the CIPN [21,22,23,24,25]. Nevertheless, as mentioned previously, this problem is still not entirely understood, and consequently, firm observations concerning the exact mechanisms of CIPN cannot be formulated.

CIPN is a current and important issue that has to be considered both before and during the administration of any anti-cancer therapy. In more severe cases, CIPN can require a dose reduction of the administered drug or even therapy termination, which consequently can decrease the rate of successful oncological treatments and survivals [5,26]. Therefore, the identification of proper treatment options for CIPN is one of the most important challenges, as ones used currently are often ineffective [27,28]. In this work, we will review dietary supplements that can potentially be used to prevent or treat CIPN.

## 2. Acetyl-L-Carnitine

Acetyl-L-carnitine (ALC), an acetyl ester of L-carnitine, present in both central and peripheral nervous systems, plays an important role in the oxidation of free fatty acids as well as in the intermediary metabolism [29,30]. Dietary supplementation of ALC exerts neuroprotective, neurotrophic, anti-depressive and analgesic effects in some painful neuropathies in animal models [31,32,33]. Furthermore, repeated ALC administration has been proven to promote regeneration of the injured nerves, increasing axon regeneration at the transected sciatic nerve stump, and restoring motor functions [29,34]. It has been proven to be effective in reducing pain in diabetes-related neuropathies, where it also seems to be capable of improving electromyography (EMG) parameters [33,35,36]. Pain reduction is probably caused by both a neuroprotective and a central anti-nociceptive mechanism of ALC [33].

Data regarding the effects of oral administration of ALC in CIPN is inconclusive, with some studies proving its ability to prevent and/or treat CIPN, while others not only prove its ineffectiveness but suggest that it might exacerbate this condition (Table 1) [30,36,37]. In the study conducted by Pisano et al. [38], focused on the animal model of cisplatin- and paclitaxel-induced neuropathy, ALC co-treatment significantly reduced the severity of sensory loss and potentiated the levels of nerve growth factor (NGF), a neuroprotective agent, thus resulting in significantly reduced severity of neuropathy [38]. Furthermore, Flatters et al. [29] have proven ALC to prevent the onset of significant, paclitaxel-related pain up to three weeks after administration of the last dose of ALC [29]. Moreover, and possibly most importantly, ALC was proven to have no effects on the antitumour activity of the cytostatic drugs [38,39].

Observations made by Ghirardi et al. [39] were consistent with those mentioned above. They argue that the co-administration of ALC was able to prevent the oxaliplatin-related neurotoxicity, assessed using both behavioural and neurophysiological methods. Moreover, it was capable of reversing some neurological damage in the follow-up period after the end of the oxaliplatin therapy [39].

Nevertheless, observations made in the clinical trials were not consistent. While Bianchi et al. [40] observed a significant reduction in the symptoms of CIPN, a study performed by Hershman et al. [37] noticed in the double-blind trial that ALC supplementation was not only ineffective in the prevention of taxane-induced neuropathy in women undergoing breast cancer therapy, but also at 24 weeks of ALC therapy an exacerbation of CIPN was observed. Interestingly, the reason behind that vastly different result is not clear [37,39].

Although ALC was somewhat capable of attenuating established mechanical hypersensitivity caused by paclitaxel and cisplatin, its efficacy as a treatment option for CIPN is disputable and inefficient in comparison to its ability to prevent the development of painful neuropathy in the first place. As mentioned previously, no study observed an increase in the incidence rate of the CIPN caused by ALC administration, while this drug might potentially result in the exacerbation of the symptoms of the CIPN [29,39].

Taking all the above studies into consideration, the application of ALC in CIPN can be justified, yet special attention should be paid to the patients with a taxane-based regime.

## 3. Vitamin B Group

All vitamins from the B group, consisting of thiamine (B1), riboflavin (B2), niacin (B3), pantothenic acid (B5), pyridoxine (B6), cobalamin (B12), folate, choline, and biotin function as coenzymes in several intermediary metabolic pathways, including neurotransmitter synthesis and neuronal membrane synthesis [41]. Deficiency of vitamins from B group, especially B12, is known to cause neuropathies, usually accompanied by paraesthesia, numbness, and ataxia [42,43,44]. Observations regarding malignancy caused by B12 deficiency are in the line with the previous statement [45]. The most important factor differentiating cancer-caused deficiency from the “normal” deficiency, is the time frame. The clinically manifested significant decrease in cobalamin intake might take from 5 to 10 years, whereas the levels of vitamin B12 in some cancer affected patients have been proven to decrease rapidly, especially during chemotherapy, and the clinical manifestations are visible already after a few months [45,46,47,48,49]. Schloss et al. [41] reported that vitamin B complex supplementation was statistically ineffective at preventing CIPN as compared to the placebo, although as indicated by the results of the Patient Neurotoxicity Questionnaire (PNQ), patients taking the vitamin B complex perceived a reduction in sensory peripheral neuropathy [41]. Importantly, in cases of CIPN coexisting with vitamin B12 deficiency, patients did benefit from the oral supplementation of this medication [41,48]. Lastly, Abe et al. [50] reported that oral vitamin B12 supplementation did not help in the prevention of the CIPN onset. Their study did not include the control group and they compared the efficacy of B12 supplementation versus goshajinkigan—observed incidence of neuropathy was 88.9% and 39.3%, respectively [50].

As described in the aforementioned studies (Table 2), vitamin B complex supplementation cannot be recommended as the main way of CIPN prevention. Nevertheless, since such therapy does not impact the effectiveness of chemotherapy (with the exception of high doses of pyridoxine), and in some particular cases could potentially have an ameliorative effect, treatment with the vitamin B complex could be a safe and cheap solution [46]. Finally, it is essential to remember that regardless of all the positive effects, vitamin B12 supplementation has been suspected to increase the risk of developing colorectal cancer [51]. Still, we believe that in this case, the benefits might outweigh the potential risks.

## 4. Vitamin E

Vitamin E is often regarded as a treatment option for several neuropathies, such as diabetic neuropathy [52,53,54]. Furthermore, it is considered a useful agent for alleviating the symptoms of other chemotherapy-related toxicities [55]. Vitamin E is proven to be a strong antioxidant capable of protecting the integrity of cellular membranes from oxidative stress; hence, it might be potentially capable to prevent the free radicals caused nerve damage [54,56,57,58]. Furthermore, although infrequent in clinical practice, vitamin E deficiency can be caused by an array of possible conditions including various cancers, such as acute lymphoblastic leukaemia, as well as treatment with chemotherapeutics, and cisplatin in particular [59,60,61,62,63,64]. Importantly, some chemotherapeutics, such as cisplatin and oxaliplatin, exhibit oxidative traits. In the case of oxaliplatin, this ability is proven to be linked to the pathogenesis of CIPN [65,66,67]. Should the observations be applied to CIPN caused by other types of chemotherapy, vitamin E could constitute a potentially excellent treatment option in this type of peripheral neuropathy.

Although the majority of data regarding the feasibility of using vitamin E as a treatment option is consistent, some significant discrepancies can be observed. Pace et al. (2003) [68] in a study focused on patients diagnosed with a variety of cancers and treated with cisplatin, discovered a positive impact of vitamin E on the incidence and severity of CIPN. Here, the incidence of neurotoxicity was significantly lower in the group simultaneously treated with vitamin E (30.7%) than it was in the group receiving placebo (85.7%). Furthermore, when measured with a comprehensive neurotoxicity score based on clinical and neurophysiological parameters (a modified neurological symptom score, grading the severity of neuropathy as 1 = mild, 2 = moderate, and more than 2 = severe), the severity of polyneuropathy was also lower in the former group—2 vs. 4.7 respectively [69]. This observation was further confirmed by several other studies conducted by: Argyriou (2005) et al. [69], Argyriou et al. [70], and Argyriou et al. [71], who all share the same observations of considerably lower incidence of CIPN in vitamin E receiving group vs. placebo group—25% vs. 73.3% [69], 21.4% vs. 68.5% [70] and 18.7% vs. 62.5% [71] respectively. The same correlation was observed in regard to the severity of CIPN, all three studies measured it with the application of the modified peripheral neuropathy (PNP) score, observing a difference between the vitamin E-receiving group and the placebo group with the results of 3.4 ± 6.3 vs. 11.5 ± 10.6 [69], 4.99 ± 1.33 vs. 10.47 ± 10.62 [70] and 2.25 ± 5.1 vs. 11 ± 11.63 [71] respectively. Pace et al. (2010) [72] continued to advocate for the use of vitamin E as a potential medication in CIPN. Their observations were consistent with all the aforementioned studies and confirmed that a group of patients treated with vitamin E exhibited lower incidence (5.9%) of CIPN as compared to a placebo group (41.7%) The severity of neurotoxicity measured with the total neuropathy score (TNS; ranging from 0 to 40, higher values indicate more severe course of neuropathy) also indicated ameliorative capabilities of vitamin E, with a mean TNS of 1.4 vs. 4.1 in vitamin E and placebo groups, respectively [72]. Lastly, Agnes et al. [73] observed vitamin E to prevent mechanical and cold allodynia caused by oxaliplatin [73].

Regardless of all the above-mentioned observations, current data is not entirely conclusive. Studies conducted by Afonseca et al. [74] and Salehi et al. [75] indicated no positive effects resulting from vitamin E administration as a preventive agent, with this discrepancy potentially resulting from different chemotherapeutics reviewed in other clinical trials or the application of different scales [74,75]. Furthermore, the study led by Kottshade et al. [76] observed that vitamin E administration did not impact the incidence rate of CIPN significantly, although as pointed out by Miao et al. [77] in their excellent meta-analysis regarding this topic, the methodology of a Kottschade et al. [76] study remains disputable, therefore any conclusions should be made with special care [75,77]. Another study conducted by Heiba et al. [78] further displays the discrepancies between particular studies. Their clinical study indicated that vitamin E supplementation neither decreased the incidence of grade 2 or above neuropathy, nor the neuropathy onset time, yet the duration of the neuropathy was clearly different depending on the placebo group and vitamin E group, 12.5 weeks vs. 5 weeks, respectively [78].

It is important to remember that even though vitamin E supplementation can be indicated to be an effective way of preventing CIPN, such treatment is not entirely safe. A prospective, multicentre clinical trial involving 35,533 men observed a 17% increased risk of developing prostate cancer after a long-time vitamin E supplementation, thus, in males, the risks of such therapy might outweigh the potential gain [79].

Taking all the previous studies (Table 3) into consideration, it can be stated that there is a strong rationale behind using vitamin E as a both preventive and treatment option in CIPN. As for now, such therapy cannot be firmly recommended and further high-quality trials with more standardized ways of reporting should be continued.

## 5. Medicinal Plants

Since ancient times, medicinal plants and herbs were used to ameliorate symptoms of a wide variety of different diseases and symptoms, including pain of different kinds, and neurological conditions [80,81,82,83]. It is believed that there is a strong rationale for this kind of medication, thus a more detailed review of its medical potential in CIPN treatment can be advised.

### 5.1. Goshajinkigan

Goshajinkigan (GJG) is a traditional Japanese medicine (Kampo) composed of ten herbs (Rehmanniae, Achyranthis Radix, Corni Fructus, Dioscoreae Rhizoma, Plantaginis Semen, Alismatis Rhizoma, Poria, Moutan Cortex, Cinnamomi Cortex, and Processi Aconiti Radix) mixed in a fixed proportion [84,85,86]. In Japan, GJG is often prescribed as a treatment option used to alleviate the symptoms of diabetic peripheral neuropathy i.e., numbness, cold sensations, and paraesthesia/dysesthesia [87,88,89,90].

An animal study conducted by Mizuno et al. [91] decided to focus on the TRP channels including Ca2^+^-permeable nonselective cation channels suggested to serve as thermal, chemical, and mechanical sensors, with a particular focus on TRPA1 and TRPM8 [92,93,94,95,96]. As observed in the real-time polymerase chain reaction (rtPCR), oxaliplatin increased the expression levels of TRPA1 and TRPM8 mRNA resulting in hypersensitivity to cold, while GJG administration prevented that increase [92]. Another animal study was performed by Ushio et al. [97], where the authors discovered that GJG was capable of preventing oxaliplatin-related cold hyperalgesia, although it had no effect on oxaliplatin-related allodynia. Importantly, the authors discovered that GJG had no negative effect on oxaliplatin-induced tumour cytotoxicity [97].

Nishioka et al. [98] sought to investigate the possibility of using GJG as a preventive option for CIPN. A group of patients diagnosed with colorectal cancer treated with a modified FOLFOX6 regime, containing oxaliplatin, was divided into two subgroups, with one subgroup receiving oral administration of GJG every day, and the other receiving placebo. Neuropathy was assessed using the Neurotoxicity Criteria of Debiopharm (DEB-NTC) during every course. Their study claimed that the incidence of grade 3 peripheral neuropathy in the GJG group was significantly lower than in the control group, and after 10 courses of chemotherapy, there were no cases of adverse effects in the study group (0%), while in the placebo-receiving group this number reached 12% (*p* < 0.01) [98]. These observations were further confirmed in the subsequent studies. Kono et al. [87,99] focused on a similar group of patients diagnosed with colorectal cancer and treated with FOLFOX regime and subdivided similarly to the previous study. Consistently with the research of Nishioka et al., Kono et al. [99] found GJG to both decrease the incidence rates of the CIPN, as measured with DEB-NTC, and ameliorate its effects [100]. Kono et al. later [87] used a different scale, Common Terminology Criteria for Adverse Events (CTC-AE), but the results remained consistent with the previous ones. Here, the incidence rate of grade 2 or greater CIPN, until the 8th cycle of chemotherapy, was 39% in the GJG group and 51% in the placebo group, with the incidence rate of grade 3 CIPN being 7% in the GJG group vs. 13% in the placebo group [87]. The last study focused on the FOLFOX6 treated group of patients diagnosed with colorectal cancer was the one performed by Oki et al. [100] The patient division introduced in the study, was similar to the division presented in the previous two studies. Importantly, this study contradicted the first two, where authors, using the scale of National Cancer Institute Common Terminology Criteria for Adverse Events (NCI CTC) to assess neuropathy, observed that GJG not only did not prevent CIPN, but it might have had a contrary effect. The incidence of grade 2 or greater neurotoxicity was 50.6% and 31.2% in the GJG and the placebo group respectively [100]. The reason for this difference remains unclear, although it might be attributed to the differences in the methodology applied in these studies. Nevertheless, this question remains yet to be answered.

Studies performed by Abe et al. [50] and Kawabata et al. [101] decided to focus on a different approach, with the main focus being the efficacy of GJG in breast cancer patients suffering from CIPN and treated with docetaxel (in the case of Abe et al.) or paclitaxel (in a case of Kawabata et al.) [50,101]. Abe et al. [50] compared the efficacy of B12 supplementation to GJG supplementation with neuropathy being evaluated according to DEB-NTC, NCI-CTC ver. 3.0, and a visual analogue scale (VAS). The authors observed an incidence of neuropathy of 39.3% in the GJG group and 88.9% in the B12 group, with a significantly lower incidence rate of adverse events in the former group [50]. Kawabata et al. [101] measured the difference in the reduction of CIPN with several questionnaires, as well as CTC-AE v4.0, and their results contradicted some of the previous studies. Here, all patients experienced CIPN of either hands or feet at 4 weeks of study and the entire GJG group experienced CIPN of both hands and feet at 12 weeks, while in the control group only 2 out of 6 patients experienced such condition at this time frame [101]. Nevertheless, it is important to mention the differences between the two studies. Abe at al. [50] study included no control group, while the group of patients being reviewed in the Kawabata et al. [101] study was small, with the GJG group consisting of only 4 patients. Furthermore, both studies used different methods of evaluating the severity of CIPN (Table 4). The discrepancy in the results between these two studies might be caused by either those factors or a sum of all of them. Nevertheless, we should be careful in drawing any conclusions.

Taking all the aforementioned studies into consideration, we believe there is a strong rationale for the application of GJG as a CIPN-preventing agent. Nevertheless, it is important to mention that not only the above-mentioned studies were inconclusive, but all of them were also performed on a Japanese population. As differences in the pharmacokinetics of the drugs between different races and ethnicities are confirmed, studies focused on other populations are required [102,103]. Lastly, it is important to mention the paucity of data regarding the overall safety of the GJG administration [104]. Taking all the aforementioned data into account, we believe that although potentially effective, therapy with GJG might cause other, yet to be described, complications.

### 5.2. Citrullus colocynthis

*Citrullus colocynthis*, also known as bitter apple, is a plant used in traditional medicine as an anti-inflammatory, antidiabetic, analgesic, hair growth-promoting, abortifacient, and antiepileptic compound with disputable results [109,110,111,112]. In Rostami et al. study focusing on patients diagnosed with CIPN, the effects of treatment with *C. colocynthis* extract oil were evaluated in the randomized, double-blind, placebo-controlled clinical trial. Unfortunately, *C. colocynthis* extract oil did not ameliorate the symptoms of CIPN and, as such, we cannot recommend such therapy [113].

### 5.3. Matricaria chamomilla

*Matricaria chamomilla* L. extract contains terpenoids like α-bisabolol and its oxide azulenes, including chamazulene and acetylene derivatives and flavonoids: apigenin, luteolin or others [114]. Apigenin was reported to have antioxidative effects, resulting in neuroprotective effects against oxidative stress in neurological disorders [110,115]. Moreover, apigenin treatment by modulating levels of cytokines and nitric oxide can be protective for neurites and cell viability against inflammation [116,117,118]. In mice models of cisplatin-induced neuropathy, *M. chamomilla* was able to decrease pain and inflammation which was measured in the formalin test [119]. To the best of authors’ knowledge, no clinical trials investigating the feasibility of using *M. chamomilla* as an ameliorative factor in CIPN have been performed to this day.

### 5.4. Salvia officinalis

*Salvia officinalis*, which contains phenolic acid and flavonoid content, has anti-inflammatory and antioxidant effects on lipopolysaccharide-induced inflammation as shown in the mice model. The *Salvia officinalis* administration resulted in a decrease of inflammatory markers, which, according to the authors’ suggestion, might be caused by the inhibition of reactive lipid peroxidation or/and the consumption of antioxidants by scavenging reactive oxygen radicals [120]. This plant can be also useful in the enhancement of cognitive activity and protection against neurodegenerative diseases [121]. In Alzheimer’s Disease, *S. officinalis* improved cognitive functions with no side effects compared to the placebo group [122,123]. The influence of *S. officinalis* on CIPN has been shown in a mice model, where its extract increased vincristine-induced pain response [124].

### 5.5. Cinnamomum cassia

*Cinnamomum cassia*, which contains coumarins, cinnamic acid, as well as cinnamaldehyde, was described as a neuroinflammation-inhibiting compound, capable of exerting its action through attenuation of iNOS, COX-2 expression and NF-κB [125]. The study by Kim et al. [126] presented that the administration of *Cinnamomum Cortex* (the bark of *C. cassia*) water extract could induce a significant suppression of the activation of astrocytes and microglia. After cold allodynia causing oxaliplatin injection, *C. Cortex* water extract has been observed to decrease the expression levels of IL-1β and TNF in the spinal cord. Moreover, the same study showed that coumarins, the compound of *C. Cortex*, can attenuate oxaliplatin-induced cold allodynia in rats [126].

### 5.6. Curcumin

Curcumin is a commonly known substance, which positively impacts our health by decreasing the risk for several pathologies: cardiovascular diseases [127], type 2 diabetes mellitus [128], cancer [129], but also against neurodegenerative disorders like Alzheimer’s disease, Parkinson’s disease, and multiple sclerosis among others [130]. Recent studies have shown that curcumin, with its poor water solubility, cannot act directly on the central nervous system, rather affecting the “microbiota-gut-brain axis”, so that the functions of the brain are preserved [131]. Curcumin, due to its influence on NF-ĸB [132], COX-2 and pro-inflammatory cytokines [133], is also described as an anti-inflammatory, antioxidant, and neuroprotective substance [134]. Curcumin, in the mice model, decreases vincristine-induced neuropathic pain including hyperalgesia and allodynia. Additionally, an antioxidative effect has been observed in the curcumin-treated group [135]. Agthong et al. [136] noticed that curcumin prevented thermal hyperalgesia in rats with cisplatin-induced neuropathy. Moreover, in this study morphometric analysis of L4 dorsal root ganglia was performed, and in the curcumin-treated group less pathological changes, such as nuclear or nucleolar atrophies including the loss of neurons, have been observed [137]. As mentioned previously, one of the pathomechanisms of cisplatin-related neuropathy is oxidative stress caused by this chemotherapeutic. As such, we can speculate that the amelioration of symptoms observed by the Agthong et al. [136] might be related to the aforementioned antioxidative traits of curcumin [137,138]. As curcumin was observed to be an overall safe compound, we believe that, in some cases, such therapy could be a cheap and safe way to ameliorate the symptoms of CIPN (Table 5) [139].

## 6. Docosahexaenoic Acid and α-Lipoic Acid

Docosahexaenoic acid (DHA) is one of the 22 carbon omega-3 fatty acids, proven to be crucial for the proper functioning of the nigrostriatal pathway, as well as exerting antinociceptive and antiapoptotic effects, all of which is crucial for the maintaining of brain function. Furthermore, DHA deficiency during foetal development can have consequences in cognitive abilities after birth, as this compound has been observed to be crucial for proper neurogenesis [140,141,142].

α-Lipoic acid (ALA) is an antioxidant, playing a key role in mitochondrial dehydrogenase reactions [143], therefore its role in brain ischemia and reperfusion injury [144] or neurodegeneration [145] has been described.

Maschio et al. [146] observed that DHA and ALA prevent and reduce the severity of bortezomib-related peripheral neuropathy in patients with multiple myeloma. Seventeen out of eighteen patients did not report any pain-related symptoms during the course of the study [146]. Dinicola et al. [147] reported that although ALA can be useful especially in CIPN patients treated with platinum compounds, in the taxane-treated group no benefits of ALA-supplementation have been observed [147]. One of the supplements, composed of ALA, *Boswellia serrata*, methylsulfonylmethane, and bromelain, was announced to reduce CIPN pain with no other toxicity, nor interactions [148]. Lastly, as observed in the Agnes et al. [73] study, orally administrated ALA might prevent mechanical and cold allodynia, a symptom of oxaliplatin-induced peripheral neuropathy [73].

DHA can be useful in diabetic neuropathy, as it can decrease dorsal root ganglia excitability and, consequently, inhibit allodynia and thermal hyperalgesia in streptozotocin (STZ)-induced diabetes rats [149,150]. Nevertheless, although we can speculate that similar observations could be made regarding CIPN, this effect is yet to be investigated. Not all of the research has proven the positive impact of DHA and ALA on CIPN. Guo et al. [151] did not observe any differences in the cisplatin- or oxaliplatin-induced CIPN severity in the oral ALA administration group compared to the placebo group [151].

Taking all of the aforementioned studies into consideration, although we believe that DHA and ALA supplementation might be useful in the CIPN treatment, this topic requires further investigation.

## 7. Sirtuin

Sirtuin 2 (SIRT2) is an NAD+-dependent histone deacetylase (HDACs) that plays a key role in many biological processes [152]. A recent study has shown that SIRT2 levels are higher in the central nervous system, particularly in the cortex, striatum, hippocampus, and spinal cord [153]. The severity of neurological diseases, such as Alzheimer’s Disease or Parkinson’s Disease was described to have correlated with SIRT2 levels, which is further associated with NAD+ metabolism [154,155,156,157]. It was also reported that SIRT2 and other sirtuins can be useful in the prevention and reversal of diabetic peripheral neuropathy symptoms [158,159,160].

Recent studies confirmed the overexpression of SIRT2 to be useful in the treatment of cisplatin-induced CIPN, although the exact mechanism is still not clearly understood. Some evidence states that SIRT2 accumulates in the nuclei of dorsal root ganglion sensory neurons and the protection of peripheral neurons against cisplatin is possible through its activation [161]. Zhao et al. [162] described that SIRT2 may have an impact on the pathways like MAPK, TNF, or the cytokine-cytokine interaction, which was a result of RNAseq technique usage in cultured rodent neurons. Cisplatin-induced changes were proven to depend on SIRT2 status, with 783 affected genes measured in SIRT2-deficient cells and 227 affected genes in the SIRT-2-expressing cells, therefore indicating SIRT2 presence to regulate the response to cisplatin [162]. To the best of authors’ knowledge, no clinical trials investigating the feasibility of using SIRT2 as an ameliorative factor in CIPN have been performed to this day.

Lastly, it is important to mention that a diet composed of “sirtuin-activating foods” might play a role in ameliorating symptoms of several chronic diseases and, as we suspect, CIPN [163]. Nevertheless, as available literature suggests, to this day no clinical trials have confirmed this belief. Considering that cancer patients are prone to malnourishment, any major diet changes should be carefully investigated, especially considering the paucity of data regarding the safety of a diet composed of “sirtuin-activating foods” [164]. Taking all of the above-mentioned information into consideration, we believe that although such a diet might seem to be an attractive opportunity for patients to easily alleviate their symptoms without any additional medications, we cannot recommend this approach.

## 8. Discussion

Chemotherapy-induced peripheral neuropathy is one of the most commonly occurring adverse effects of chemotherapy, afflicting a high percentage of patients undergoing cancer treatment.

Multiple studies offer compelling evidence concerning the efficacy of naturally occurring substances and compositions both in CIPN prevention and treatment. However, the tolerance, efficacy, and safety profile of various substances mentioned may vary significantly. Among others, acetyl-L-carnitine seems to be an evidence-based option, with several studies proving its efficacy, acceptable safety profile, and no detected interactions with drugs used in cancer chemotherapy.

Given the relatively high number of substances with a peptide structure or classified as fats, considered as perspective options in CIPN treatment or prevention, perhaps it can be argued that apart from supplementation, general dietary management, including the use of a diet rich in specific substances, may also be appropriate.

Some evidence supports the opinion that docosahexaenoic acid, α-lipoic acid and SIRT2 may produce positive effects on CIPN management. The same applies to vitamins. In the case of vitamin supplementation, vitamins B and E have the broadest support in the literature. The effectiveness of group B vitamins, although their supplementation seems to be overall safe and cheap, can be however seen as questionable. The research on vitamin E, on the other hand, shows promising results, but at least in the case of prostate cancer, supplementation might worsen the overall prognosis, and should therefore be carried out with extreme caution.

Including the evidence from randomized trials, some other evidence may support the idea that plant-based products, including substances found in plants traditionally considered medicinal in some parts of the world, might be beneficial. Particularly goshajinkigan should be mentioned, even if its efficacy is not confirmed. Furthermore, *Citrullus colocynthis* might be an interesting option to research as this traditionally used medicinal plant has been poorly investigated in CIPN. Importantly, recent papers reveal that there are many more plants yet to be investigated, that could potentially be beneficial to CIPN patients. Atractylenolides, isolated from the rhizomes of Atractylodes species, as well as Mist Antiaris, a monoherbal decoction prepared from the stem bark of *Antiaris Africana*, could be examples of such agents. The former two has been observed to possess neuroprotective, antioxidative and anti-inflammatory traits, thus making them a potentially interesting option to be reviewed in the future [165]. The effects of the latter are less understood, nevertheless it has been observed to alleviate symptoms of neuropathy [166]. As such, investigating the ability of those compounds to alleviate the symptoms of CIPN could be an interesting opportunity to discover new treatment options.

## 9. Conclusions

As the survival rates of cancer improve, more patients are affected by the CIPN, thus leading us to evaluate the new therapeutic options. In our study, we reviewed lesser-known drugs used in the CIPN therapy. While some of them are promising and others are remaining to be ineffective or even potentially harming to patients, none of them was fully effective in either treatment or prevention. The currently used treatment is suboptimal, frequently resulting in a forcibly decreased dosage of chemotherapeutics and often requiring the termination of chemotherapy altogether. As a plethora of clinical trials are being conducted, a new hope for the affected patients arises. We are of the opinion that further clinical studies concerning new or rarely applied options in CIPN therapy may contribute to the development of oral supplementation-based treatment as an alternative or improvement of the current pharmacotherapy options in CIPN patients.

## Figures and Tables

**Table 1:** Efficacy of ALC in CIPN.

Study	Types of Study	*n*	Chemotherapy	Results
Pisano et al. [38]	Preclinical Animal	n.a.	Cisplatin	Reduced severity of CIPN in the animal model
Flatters et al. [29]	Preclinical Animal	n.a.	Paclitaxel	Decreased NIR Reduced severity of CIPN in the animal model
Ghirardi et al. [39]	Preclinical Animal	n.a.	Oxaliplatin	Decreased NIR Ability to reverse neurological damage
Bianchi et al. [40]	Clinical	25	Paclitaxel	Ameliorated CIPN symptoms
Hershman et al. [37]	Clinical	409	Paclitaxel	No effect on NIR Exacerbation of CIPN at 24 weeks of ALC administration

n.a.—not applicable; ALC—acetyl-L-carnitine; CIPN—chemotherapy-induced peripheral neuropathy; NIR—neuropathy incidence rate.

**Table 2:** Efficacy of vitamin B group in CIPN.

Study	Type of Study	*n*	Chemotherapy	Results
Schloss et al. (2017) [41]	Clinical	71	Taxanes Oxaliplatin Vincristine	No effect on NIR Patient perceived reduction in sensory peripheral neuropathy (PNQ)
Abe et al. [50]	Clinical	70	Docetaxel	Worse NIR as compared to goshajinkigan supplementation

CIPN—chemotherapy-induced peripheral neuropathy; NIR—neuropathy incidence rate; PNQ—Patient Neurotoxicity Questionnaire.

**Table 3:** Efficacy of vitamin E in CIPN.

Study	Type of Study	*n*	Chemotherapy	Results
Agnes et al. [73]	Preclinical Animal	n.a.	Oxaliplatin	Reduced severity of CIPN in the animal model
Pace et al. (2003) [68]	Clinical	27	Cisplatin	Decreased NIR
Argyriou et al. (2005) [69]	Clinical	31	Cisplatin Paclitaxel	Decreased NIR Reduced severity of CIPN
Argyriou et al. (2006) [70]	Clinical	30	Cisplatin	Decreased NIR Reduced severity of CIPN
Argyriou et al. (2006) [71]	Clinical	32	Paclitaxel	Decreased NIR Reduced severity of CIPN
Kottschade et al. [76]	Clinical	189	Taxane Cisplatin Carboplatin Oxaliplatin	No effect on NIR
Pace et al. (2010) [72]	Clinical	41	Cisplatin	Decreased NIR Reduced severity of CIPN
Afonseca et al. [74]	Clinical	34	Oxaliplatin	No effect on NIR
Salehi et al. [75]	Clinical	65	Oxaliplatin	No effect on NIR
Heiba et al. [78]	Clinical	140	Paclitaxel	No effect on NIR Shortened duration of CIPN

n.a.—not applicable; NIR—neuropathy incidence rate; CIPN—chemotherapy-induced peripheral neuropathy.

**Table 4:** Comparison of different neuropathy assessing scales.

Scale	Grade 0	Grade 1	Grade 2	Grade 3	Grade 4	Grade 5
Eastern Clinical Oncology Group (ECOG) [105]	No symptoms	Mild paraesthesia, loss of deep tendon reflexes	Severe paraesthesia, mild or moderate objective sensory loss	Paraesthesia interfering with functioning, severe objective sensory loss	n.a.	n.a.
National Cancer Information Center—Common Toxicity Criteria (NCI-CTC) [106]	No symptoms	Mild paraesthesia, loss of deep tendon reflexes	Moderate paraesthesia, mild or moderate objective sensory loss	Sensory loss, paraesthesia interfering with functioning	n.a.	n.a.
National Cancer Institute Common Terminology Criteria for Adverse Events (CTC-AE) [107]	n.a.	Asymptomatic; mild paraesthesia, loss of deep tendon function	Sensory alteration or paraesthesia interfering with function, but not with ADL	Sensory alteration or paraesthesia interfering with ADL	Disability	Death
Neurotoxicity Criteria of Debiopharm (DEB-NTC) [108]	n.a.	Duration of less than 7 days	Duration of more than 7 days	Impairment of function interfering with ADL	n.a.	n.a.
Visual Analogue Scale (VAS)	Visual scale indicating the severity of symptoms					
Total Neuropathy Score (TNS) [109]	Minimum score of 0, maximum score of 40 10 different evaluated factors Higher values indicate more severe course of neuropathy					

n.a.—not applicable; ADL—activities of daily life

**Table 5:** Efficacy of medicinal plants and herbal medicines in CIPN.

Study	Type of Study	*n*	Reviewed Agent	Chemotherapy	Results
Nishioka et al. [98]	Clinical	45	Goshajinkigan	Oxaliplatin	Decreased NIR No effect on anti-tumour activity of oxaliplatin
Kono et al. (2011) [99]	Clinical	55	Goshajinkigan	Oxaliplatin	Decreased NIR Decrease grade 3 CIPN
Ushio et al. [97]	Preclinical Animal	n.a.	Goshajinkigan	Oxaliplatin	GJG ameliorates CIPN symptoms in animal model No effect on anti-tumour activity of oxaliplatin
Kono et al. (2013) [87]	Clinical	89	Goshajinkigan	Oxaliplatin	Decreased NIR No effect on anti-tumour activity of oxaliplatin
Abe et al. [50]	Clinical	60	Goshajinkigan	Docataxel	Decreased NIR
Kawabata et al. [101]	Clinical	18	Goshajinkigan	Paclitaxel	Increased NIR
Mizuno et al. [91]	Preclinical Animal	n.a.	Goshajinkigan	Oxaliplatin	GJG ameliorates CIPN symptoms in animal model
Oki et al. [100]	Clinical	183	Goshajinkigan	Oxaliplatin	No effect on NIR
Rostami et al. [113]	Clinical	34	*Citrullus colocynthis*	Not determined	No improvement in CIPN symptoms
Abad et al. (2011) [119]	Preclinical Animal	n.a.	*Matricaria chamomilla*	Cisplatin	Chamomile ameliorates CIPN symptoms in animal model
Abad et al. (2011) [124]	Preclinical Animal	n.a.	*Salvia officinalis*	Vincristine	Salvia ameliorates CIPN symptoms in animal model
Babu et al. [135]	Preclinical Animal	n.a.	Curcumin	Vincristine	GJG ameliorates CIPN symptoms in animal model
Agthong et al. [136]	Preclinical Animal	n.a.	Curcumin	Cisplatin	GJG ameliorates CIPN symptoms in animal model

n.a.—not applicable; CIPN—chemotherapy-induced peripheral neuropathy; NIR—neuropathy incidence rate; GJG—goshajinkigan.

## Data Availability

Not applicable.
